# Inpatient hospital complications and lengths of stay: a short report

**DOI:** 10.1186/1756-0500-4-135

**Published:** 2011-05-05

**Authors:** Ronald J Lagoe, Pamela E Johnson, Mark P Murphy

**Affiliations:** 1 Hospital Executive Council, PO Box 35089, Syracuse, New York, 13235, USA; 2 Community General Hospital, Broad Road, Syracuse, New York, 13215, USA; 3 St. Joseph's Hospital Health Center, 301 Prospect Avenue, Syracuse, New York, 13203, USA

## Abstract

**Background:**

Increasingly, efforts are being made to link health care outcomes with more efficient use of resources. The current difficult economic times and health care reform efforts provide incentives for specific efforts in this area.

**Findings:**

This study defined relationships between inpatient complications for urinary tract infection and pneumonia and hospital lengths of stay in three general hospitals in the metropolitan area of Syracuse, New York. It employed the Potentially Preventable Complications (PPC) software developed by 3M™ Health Information Services to identify lengths of stay for patients with and without urinary tract infection and pneumonia. The patient populations included individuals assigned to the same All Patients Refined Diagnosis Related Groups and severity of illness. The comparisons involved two nine month periods in 2008 and 2009.

The study demonstrated that patients who experienced the complications had substantially longer inpatient hospital stays than those who did not. Patients with a PPC of urinary tract infection stayed a mean of 8.9 - 11.9 days or 161 - 216 percent longer than those who did not for the two time periods. This increased stay produced 2,020 - 2,427 additional patient days.

The study demonstrated that patients who experienced the complications had substantially longer inpatient hospital stays than those who did not. Patients with a PPC of pneumonia stayed a mean of 13.0 - 16.3 days or 232 - 281 percent longer than those who did not for the two time periods. This increased stay produced 2,626 - 3,456 additional patient days. Similar differences were generated for median lengths of stay.

**Conclusions:**

The differences in hospital stays for patients in the same APR DRGs and severity of illness with and without urinary tract infection and pneumonia in the Syracuse hospitals were substantial. The additional utilization for these complications was valued at between $2,000,000 - $3,000,000 for a three month period. These differences in the use of hospital resources have important implications for reduction of health care costs among providers and payors of care.

## Findings

In recent years, interest in the improvement of quality within the United States health care industry has increased. One of the most important aspects of this development is recognition that better health care outcomes are related to more efficient use of resources [1,2]. This movement should stimulate much needed change in an industry which historically has voiced support for improving quality but has sometimes experienced difficulty bringing this about.

Research concerning the connection between improved outcomes and more efficient resource use is increasing interest in this topic among the leadership of this sector. Such research is helping individual hospitals reduce costs during difficult economic times, as well as preparing them for financial disincentives related to complications and readmissions developed by government and private payors [3,4].

This study concerned the relationship between inpatient complications and lengths of stay for urinary tract infection and pneumonia in the general hospitals of the metropolitan area of Syracuse, New York. These acute care facilities (2010 discharges excluding newborns in parentheses) include Community General Hospital of Greater Syracuse (7,373), Crouse Hospital (20,338), and St. Joseph's Hospital Health Center (22,421). The area also includes University Hospital of the State University of New York Upstate Medical University (19,655), which did not participate in the program.

Historically, these hospitals have worked cooperatively to develop programs to improve efficiency and outcomes through the Hospital Executive Council [5,6]. As an extension of previous efforts to improve the efficiency of care, the Syracuse hospitals and the Hospital Executive Council became involved in an evaluation of the Potentially Preventable Complications (PPC) software developed by 3M™ Health Information Systems.

## Method

Potentially Preventable Complications is a system for categorizing and evaluating inpatient hospital complications. The complications included in the system were identified through a review of existing literature, the diagnostic codes used in the Complications Screening Protocol, and the Patient Safety Indicators developed by the Agency for Health Care Research and Quality by the 3M™ Corporation [7,8].

Under the Potentially Preventable Complications System a number of diagnoses are excludes as non preventable. These include complications directly related to malignant diseases, multiple trauma, organ transplants, specific burns, HIV related disorders, and neonates. Additional diagnoses are excluded only when accompanied by another specific diagnosis [9].

Candidate diagnoses that are not excluded are identified as present or not present on hospital admission. The Present on Admission Indicator is applied to each secondary diagnosis of each hospital inpatient. Secondary diagnoses identified as not present on admission are candidates for complications [10,9].

Studies completed by 3M™ Health Information Systems using administrative data bases have suggested that patients who experience inpatient complications use considerably more resources on average than those who do not These studies were based on estimated hospital charges, rather than actual utilization [11].

This case study employed actual utilization data for selected Potentially Preventable Complications in the Syracuse hospitals to identify the impact of diagnoses on inpatient utilization in the Syracuse hospitals, especially inpatient discharges and lengths of stay. The study involved data for Potentially Preventable Complications for urinary tract infection and pneumonia generated by inpatients at Community-General Hospital of Greater Syracuse, Crouse Hospital, and St. Joseph's Hospital Health Center.

This study was related to a project involving the development of interventions to prevent inpatient complications in hospitals. The interventions were implemented in October 2008. In order to control for the impact of the demonstration program in Syracuse, the data concerned inpatients during the periods January - September 2008 and 2009, the nine months preceding and following implementation of the program interventions.

The study employed a simple design comparing hospital inpatient lengths of stay and discharges for patients experiencing each of the two complications with stays and discharges for those who did not. The comparison involved PPC patients and patients who did not experience each PPC assigned to the same All Patients Refined Diagnosis Related Groups and Severity of Illness Categories.

The All Patients Refined Diagnosis Related Groups were developed by 3M™ Health Information Systems to describe and evaluate the utilization and outcomes of a full range of hospital inpatients including all ages and payor statuses. The All Patients Refined Diagnosis Related Groups are driven by the principal diagnosis or procedure of each patient.

The Potentially Preventable Complications System employs principal diagnoses, secondary diagnoses, age, and other factors to determine Severity of Illness. The use of these variables makes it possible to identify Severity of Illness based on the condition of the whole patient, rather than the principal diagnosis only.

In this study of hospital of hospital stays and discharges, inpatient discharges were assigned to comparison groups based on the same All Patients Refined Diagnosis Related Groups and Severity of Illness. This design did not account for every clinical and demographic variable that could have involved these populations. At the same time, it controlled for the impact of diagnoses which could have caused differences in stays without regard to inpatient complications.

## Results

Data for urinary tract infection, the PPC with the highest frequency in the Syracuse hospitals, are summarized in Table 1. This information demonstrated that between January and September 2008, 227 discharges in the three hospitals experienced urinary tract infections, compared with 5,877 discharges in the same APR DRGs and severity of illness levels who did not experience this complication. This amounted to a 3.9 percent complication rate. Between January and September 2009, there were 204 discharges with the PPC and 5,182 patients at risk, also resulting in a complication rate of 3.9 percent.

**Table 1:** Hospital lengths of stay and complications medical/surgical patients by APR DRG and severity of illness major PPC 16 - urinary tract infection Syracuse Hospitals January - September 2008 - 2009

	Patients with PPC				Patients at Risk without PPC				Excess Days for PPC Patients
	Number of Discharges	Patient Days	Mean LOS Per Discharge	Median LOS Per Discharge	Number of Discharges	Patient Days	Mean LOS Per Discharge	Median LOS Per Discharge	
January - September 2008									
Community General	52	564	10.8	10.0	1,359	5,938	4.4	3.6	332.8
Hospital									
Crouse Hospital	67	1,158	17.3	11.2	1,414	7,659	4.4	5.3	864.3
St. Joseph's Hospital	108	1,548	14.3	12.0	3,104	18,514	6.0	4.8	896.4
Health Center									
Total	227	3,270	14.4	10.4	5,877	32,111	5.5	4.4	2,020.3
January - September 2009									
Community General	29	485	16.7	15.0	664	3,260	4.9	3.8	342.2
Hospital									
Crouse Hospital	80	1,224	15.3	9.5	1,486	7,733	5.2	4.7	808.0
St. Joseph's Hospital	95	1,837	19.3	17.0	3,032	17,672	5.8	5.1	1,282.5
Health Center									
Total	204	3,546	17.4	14.0	5,182	28,665	5.5	4.8	2,427.6

For mean lengths of stay comparisons within individual hospitals p = .05.

Medical/Surgical exclude rehabilitation.

Sources: Hospital Executive Council; 3M Health Information Systems.

The data identified substantial differences in mean lengths of stay between patients identified with urinary tract infection as a potentially preventable complication and those without it. These differences within individual hospitals were significant at the .05 level. Compared with patients in the same APR DRGs and severity of illness levels, discharges with the complication in the three hospitals combined in January - September 2008 stayed an average of 8.9 days or 161.8 percent longer, while discharges with the PPC in January - September 2009 stayed 11.9 days or 216.4 percent longer. Individual hospital differences ranged from 145.4 to 293.2 percent longer in 2008 and from 194.2 to 240.8 percent longer in 2009.

The data in Table 1 also demonstrated that, between January and September 2008, the PPC population produced 2,020 additional patient days, or an excess average daily census of 7.4 patients. Between January and September 2009, the PPC population generated 2,427 additional days, or an excess average daily census of 8.9 patients. At a conservative late stay rate of $500 per day, this excess hospitalization was valued at between $1,010,000 and $1,213,500 for the nine month period. The reduction of even a portion of the inpatient complications responsible for this excess hospitalization could result in significant savings.

Median stay data for urinary tract infection for the two study periods are summarized in Table 1. Between January and September 2008, median length of stay for patients with this complication was 6.0 days, or 136.4 percent longer for the discharges with complications, while between January and September 2009, the median stay was 9.2 days, or 191.7 percent longer than the stay for uncomplicated discharges.

The same comparisons of utilization for patients with and without complications was applied to community acquired pneumonia. Data concerning general utilization and mean lengths of stay are summarized in Table 2.

**Table 2:** Hospital lengths of stay and complications medical/surgical patients by APR DRG and severity of illness major PPC 04 - pneumonia & other lung infections Syracuse Hospitals January - September 2008 - 2009

	Patients with PPC				Patients at Risk without PPC				Excess Days for PPC Patients
	Number of Discharges	Patient Days	Mean LOS Per Discharge	Median LOS Per Discharge	Number of Discharges	Patient Days	Mean LOS Per Discharge	Median LOS Per Discharge	
January - September 2008									
Community General	33	372	11.3	10.7	728	3,598	4.9	4.7	211.2
Hospital									
Crouse Hospital	31	481	15.5	9.5	617	3,568	5.8	4.8	300.7
St. Joseph's Hospital	148	3,827	25.9	24.0	3,073	18,279	5.9	5.4	2,960.0
Health Center									
Total	212	4,680	22.1	16.0	4,418	25,445	5.8	5.1	3,455.6
January - September 2009									
Community General	34	515	15.1	12.5	679	3,347	4.9	3.9	346.8
Hospital									
Crouse Hospital	53	982	18.5	16.5	1,076	5,679	5.3	5.2	699.6
St. Joseph's Hospital	115	2,256	19.6	15.5	2,750	16,015	5.8	5.5	1,587.0
Health Center									
Total	202	3,753	18.6	14.0	4,505	25,041	5.6	5.1	2,626.0

University Hospital data not included because of coding problems identified in 2008 data.

Medical/Surgical exclude rehabilitation.

Sources: Hospital Executive Council; 3M Health Information Systems.

As in the case of urinary tract infections, the data for pneumonia as a Potentially Preventable Complications identified relatively low complication rates among the Syracuse hospitals. Between January and September 2008, total of 212 inpatient discharges involved the pneumonia PPC at the combined hospitals compared with 4,418 discharges for patients with the same APR DRGs and severity of illness levels, resulting in a complication rate of 4.8 percent.

Between January and September 2009, there were 202 discharges and 4,505 patients at risk, resulting in a complication rate of 4.5 percent.

The data identified substantial differences between inpatient hospital lengths of stay for patients with and without the pneumonia Potentially Preventable Complication in the Syracuse hospitals. These differences within individual hospitals were significant at the .05 level. During the period of the study, an analysis focused on discharges in the same APR DRGs and severity of illness levels. The population that experienced the complication between January and September 2008 generated mean lengths of stay 16.3 days, or 281.0 percent longer than the one that did not, while discharges with the PPC between January and September 2009 stayed an average of 13.0 days, or 232.1 days longer. Individual hospital stays for the population with the complication ranged from 130.6 to 339.0 percent longer in 2008 and 208.2 to 249.1 percent longer in 2009.

The number of additional days associated with pneumonia between January and September 2008 was 3,455.6, or an excess average daily census of 12.7 patients. Between January and September 2009, the number of additional days was 2,626, or an excess average daily census of 9.6 patients. At a late stay rate of $500 per day, this excess utilization was valued at between $1,313,000 and $1,727,500 for the nine month period. The reduction of even a portion of the inpatient complications responsible for this excess hospitalization could result in significant savings.

Median stay data for pneumonia in the two study periods are summarized in Table 2. Between January and September 2008, the median length of stay for patients with this complication was 10.9 days, or 213.7 percent longer than those who did not. Between January and September 2009, the median stay for patients with the PPC was 8.9 days or 174.5 percent longer than those who did not.

## Discussion

In the United States, the health care reform movement and other factors are generating greater interest in the relationship between health care outcomes and efficiency. This study addressed a limited aspect of this subject, the relationship between inpatient hospital complications and lengths of stay. Through evaluation of this issue for the two most frequent inpatient complications, urinary tract infection and pneumonia, in the hospitals of Syracuse, New York, it identified large differences in lengths of stay between patients who experienced the complications and those who did not. The actual differences in stays were considerable, with patients who experienced complications usually staying two to three times as long as those without complications. The analysis of these data, based on patients assigned to the same All Patients Refined Diagnosis Related Groups and Severity of Illness categories, did not exclude all extraneous variables, however, it did control for most aspects of clinical conditions.

The analysis included data for nine month periods in 2008 and 2009, before and after a series of interventions was implemented in the fourth quarter of 2008. It was notable that, while the numbers of patients experiencing complications decreased after the implementation of interventions, the differences between lengths of stay for patients with and without complications actually increased. It may be that, after the interventions, remaining patients experienced higher severity of illness and stayed longer.

The interventions used to address urinary tract infection included protocols for the removal of urinary catheters and frequent monitoring of potential infection sites and laboratory data. For pneumonia, the interventions included early ambulation of patients, elevation of head of patient bed, and hourly use of spirometry.

The differences in hospital stays were reflected in the use of resources in the participating hospitals. For this part of the analysis, the study used actual cost data for the hospitals, rather than estimated charges. This was possible because the study directly involved the administrations of the participating hospitals. The study suggested that an average daily census of 16 to 21 patients was generated by these complications alone in the same hospitals. The additional utilization for both PPCs was valued at between $2,000,000 and $3,000,000 for a nine month period.

It should be emphasized that the primary purpose of the reduction of inpatient complications is to improve the health and treatment of individuals treated in hospitals. Avoiding these adverse events contributes significantly to the quality of life of these individuals. Reductions in hospital stays brought about by lower complication rates could also eliminate large expenditures for pharmaceuticals and testing. The impact of such reductions on nursing, a major component of hospital costs, would depend on the staffing programs of individual hospitals. For hospitals using traveling nurses, shorter stays could reduce the number of these positions that are required. For other hospitals, shorter stays could contribute to greater efficiency or permit reassignment of staff nurses. All of these reductions could generate substantial savings for hospitals and bring about decreases in health care costs. Reduction of inpatient hospital complications is a useful cost saving approach because these outcome are within the control of hospital staffs.

## Competing interests

The authors declare that they have no competing interests.

## Authors' contributions

RL was responsible for the design of the study and development of the data. MM was responsible for development of the data for St. Joseph's Hospital Health Center. PJ was responsible for development of the data for Community-General Hospital. All authors have read and approved the final manuscript.
